# Possible roles of monocytes/macrophages in response to elephant endotheliotropic herpesvirus (EEHV) infections in Asian elephants (*Elephas maximus*)

**DOI:** 10.1371/journal.pone.0222158

**Published:** 2019-09-06

**Authors:** Saralee Srivorakul, Thunyamas Guntawang, Varankpicha Kochagul, Kornravee Photichai, Tidaratt Sittisak, Thittaya Janyamethakul, Khajohnpat Boonprasert, Siripat Khammesri, Warangkhana Langkaphin, Veerasak Punyapornwithaya, Phongsakorn Chuammitri, Chatchote Thitaram, Kidsadagon Pringproa

**Affiliations:** 1 Veterinary Diagnostic Laboratory, Faculty of Veterinary Medicine, Chiang Mai University, Chiang Mai, Thailand; 2 Department of Veterinary Biosciences and Veterinary Public Health, Faculty of Veterinary Medicine, Chiang Mai University, Chiang Mai, Thailand; 3 Patara Elephant Farm, Hang Dong, Chiang Mai, Thailand; 4 Center of Excellence in Elephant and Wildlife Research, Chiang Mai University, Chiang Mai, Thailand; 5 Maesa Elephant Camp, Mae Rim, Chiang Mai, Thailand; 6 National Elephant Institute, Forest Industry Organization, Lampang, Thailand; 7 Department of Food Animal Clinic, Faculty of Veterinary Medicine, Chiang Mai University, Chiang Mai, Thailand; 8 Department of Companion Animals and Wildlife Clinics, Faculty of Veterinary Medicine, Chiang Mai University, Chiang Mai, Thailand; CEA, FRANCE

## Abstract

Elephant endotheliotropic herpesvirus-hemorrhagic disease (EEHV-HD) is the primary cause of acute, highly fatal, hemorrhagic diseases in young Asian elephants. Although monocytopenia is frequently observed in EEHV-HD cases, the role monocytes play in EEHV-disease pathogenesis is unknown. This study seeks to explain the responses of monocytes/macrophages in the pathogenesis of EEHV-HD. Samples of blood, frozen tissues, and formalin-fixed, paraffin-embedded (FFPE) tissues from EEHV1A-HD, EEHV4-HD, co-infected EEHV1A and 4-HD, and EEHV-negative calves were analyzed. Peripheral blood mononuclear cells (PBMCs) from the persistent EEHV4-infected and EEHV-negative calves were also studied. The results showed increased infiltration of Iba-1-positive macrophages in the inflamed tissues of the internal organs of elephant calves with EEHV-HD. In addition, cellular apoptosis also increased in the tissues of elephants with EEHV-HD, especially in the PBMCs, compared to the EEHV-negative control. In the PBMCs of persistent EEHV4-infected elephants, cytokine mRNA expression was high, particularly up-regulation of TNF-α and IFN-γ. Moreover, viral particles were observed in the cytoplasm of the persistent EEHV4-infected elephant monocytes. Our study demonstrated for the first time that apoptosis of the PBMCs increased in cases of EEHV-HD. Furthermore, this study showed that monocytes may serve as a vehicle for viral dissemination during EEHV infection in Asian elephants.

## Introduction

Elephant endotheliotropic herpesvirus-hemorrhagic disease (EEHV-HD) is one of the most highly fatal, infectious, elephant diseases worldwide [1–3]. In Thailand, EEHV infection accounts for an estimated over 80% or more of elephant calf fatalities each year. Eight species of EEHV, including EEHV1A, EEHV1B, and EEHV2-7, have been identified; they are collectively classified as members of the subfamily *Betaherpesvirinae*, genus *Proboscivirus* [4]. Although adult elephants also appear susceptible to EEHV, mortality from EEHV-HD has mostly been observed in young animals, especially those 1–8 years old [1, 5]. Most elephants that succumbed to EEHV-HD presented clinically with decreased thrombocytes and monocytes in their blood [5–9]. The marked reduction of peripheral blood thrombocytes (thrombocytopenia) and monocytes (monocytopenia) can, therefore, provide a prognostic hematological parameter for EEHV-HD cases [1, 5]. While thrombocytopenia in EEHV-HD cases is hypothesized to be a mechanism associated with consumption coagulopathies [5], the pathomechanisms underlying monocytopenia in EEHV-HD cases remain poorly understood.

Monocytes/macrophages play a crucial role in the host defense mechanism against pathogen infections and dissemination [10–14]. During innate immune responses, monocytes/macrophages sense and promote inflammation by producing type 1 interferon and secreting pro-inflammatory cytokines, in order to recruit effecter cells to clear pathogens [15–17]; while in adaptive immune response, they serve as antigen-presenting cells prime to T lymphocytes [18]. Experimental inoculation of monocytes with dengue virus *in vitro* increased apoptosis of virus-infected cells and induced production of several inflammatory cytokines, including tumor necrosis factor-α (TNF-α) [10, 11]. Apoptosis of monocytes/macrophages in dengue virus infection is believed to be an immunological process that inhibits virus replication in infected patients [10, 11]. However, to the best of our knowledge, no studies have investigated apoptosis of monocytes/macrophages in cases of EEHV infection.

Although previous reports have found the genome and protein of EEHV in the cytoplasm of monocytes/macrophages in Asian elephants that have died from EEHV-HD [6, 19], this could be part of the presentation of antigens in adaptive immune responses [18]. Thus, it is still unclear whether monocytes/macrophages serve as carrier cells for EEHV dissemination during acute and persistent virus infection. This study, therefore, aimed to investigate apoptosis of the monocytes/macrophages in response to acute and persistent EEHV infections; and to study the roles of monocytes/macrophages in the pathogenesis of EEHV infection in Asian elephants.

## Materials and methods

### Ethical statement

All experimental animal protocols in this study were consistent with the Guide for the Care and Use of Laboratory Animals (National Institute of Animal Health, WA), and were approved by the Institutional Animal Care and Use Committee (IACUC), Faculty of Veterinary Medicine, Chiang Mai University (permission number: S25/2560).

### Experimental design

Archived blood data of EEHV1A-HD and EEHV4-HD cases from 2015 to 2018, such as complete blood counts, obtained and analyzed within the 3 days prior to an elephant’s death, were selected from the Veterinary Diagnostic Laboratory, Faculty of Veterinary Medicine, Chiang Mai University, Thailand and included in the study. Then, the available frozen tissues or the formalin fixed, paraffin embedded (FFPE) tissues of those elephants were investigated as described below. Moreover, elephants that died due to co-infections of EEHV1A and 4-HD and elephants died due to unrelated EEHV infection (EEHV-negative) were also included in the study.

To investigate the roles of monocytes in the healthy EEHV-positive calves, blood samples were collected from the persistent EEHV4-infected and EEHV-negative calves, with infection status determined by conventional polymerase chain reaction (PCR) test during the prior six months. The details of each animal’s history and the tests performed are shown in Table 1.

**Table 1:** Summary of elephants’ history and tests performed, including post-mortem methods.

Animal no. [Ref.]	Age	Sex	Status	EEHV subtype*	Blood test	H&E	IHC	TUNEL	IFA of PBMCs	TEM	qRT-PCR^&^
1 [6,7]	3-y	F	Deceased	EEHV1A	NA	√	√	√	NA	NA	NA
2 [7]	2-y	F	Deceased	EEHV1A	√	√	√	√	NA	NA	NA
3	2-y	M	Deceased	EEHV1A	√	NA	NA	NA	NA	NA	NA
4	2-y	M	Deceased	EEHV1A	√	NA	NA	NA	NA	NA	NA
5 [6,7]	3-y	F	Deceased	EEHV4	√	√	√	√	√	NA	NA
6 [7]	2-y	F	Deceased	EEHV4	√	√	√	√	NA	NA	NA
7	6-y	F	Alive	EEHV4	NA	NA	NA	√	√	√	√
8	5-y	F	Alive	EEHV4	NA	NA	NA	√	√	√	√
9	4-y	F	Deceased	EEHV1A and 4	NA	√	√	NA	NA	NA	NA
10	6-y	F	Deceased	EEHV1A and 4	NA	√	√	NA	NA	NA	NA
11	NA	M	Deceased	Negative	NA	√	√	NA	NA	NA	NA
12 [6,7]	1-d	M	Deceased	Negative	NA	√	√	√	NA	NA	NA
13	35-y	M	Deceased	Negative	NA	√	√	NA	NA	√	NA
14	5-y	M	Alive	Negative	√	NA	NA	√	√	NA	√
15	8-y	M	Alive	Negative	√	NA	NA	√	√	NA	√

* determined by conventional PCR and gene sequencing

^&^ quantitative RT-PCR for cytokine mRNA expression

NA: Not applicable, F: female, M: male, y: year-old, d: day-old, H&E: hematoxylin and eosin stain, IHC: immunohistochemistry, TUNEL: Terminal deoxynucleotidyltransferase-mediated dUTP nick end labeling, IFA: immunofluorescence, gB: glycoprotein B, qRT-PCR: quantitative reverse transcriptase polymerase chain reaction, PBMCs: peripheral blood mononuclear cells, TEM: transmission electron microscopy

### Tissue processing and immunohistochemistry

FFPE samples for histological and immunohistochemical analyses were cut 4 μm thick and were either stained with hematoxylin and eosin (H&E), or placed on 3-Aminopropyl-triethoxysilane coated-slides for immunohistochemical tests. The tissues were grouped by EEHV status those from elephants that died from EEHV1A (n = 2), EEHV4 (n = 2), and co-infections of EEHV1A and 4 (n = 2), as well as, an EEHV-negative control group (n = 3). The immunohistochemistry of the tissues was investigated employing a minor modification of the avidin–biotin complex (ABC) method, as described previously [20]. Briefly, the FFPE sections were dewaxed, rehydrated and microwaved for 30 minutes in a citrate buffer (pH 6.0). Afterward, sections were incubated for 5 minutes with 3%H_2_O_2_ in methanol, and then blocked for 30 minutes at room temperature (RT) with phosphate buffer saline (PBS) containing 5% normal goat serum, 0.1% Tween-20. Thereafter, they were incubated at 37°C for 2 hours with one of two primary antibodies: a mouse monoclonal anti-ionized calcium binding adaptor molecule-1 (Iba-1) (1:200; EMD Millipore, CA, USA) or a rabbit polyclonal anti-EEHV gB[6]. After washing three times with PBS, sections were incubated for 45 minutes at RT with either biotinylated goat anti-mouse or goat anti-rabbit secondary antibodies (1:200; Vector Laboratories, CA, USA). Section then were washed and incubated with peroxidise coupled ABC (Thermo Fischer Scientific, Waltham, MA, USA). Antibody binding was visualized using 3,3-diaminobenzidine-tetrahydrochloride (DAB)-H_2_O_2_ for 5 minutes at RT followed by counterstaining with Mayer´s hemalum. For each antibody, a normal mouse or rabbit serum was used instead of a primary antibody. Slides were observed and photos were taken under a light microscope. Three independent observers scored the immunohistochemically positive cells for Iba-1 and EEHV gB antibodies in selected organs; samples were rated as 0 (no positive cells), 1 (1–10% positive cells), 2 (11–30% positive cells), 3 (31–50% positive cells), or 4 (>50% positive cells). The percentage of positive cells was recorded for every tissue sample.

### Isolation of the elephant’s peripheral blood mononuclear cells (PBMCs)

PBMCs were isolated from the persistent EEHV4-infected (n = 2) and EEHV-negative (n = 2) calves, as previously described [6]. Briefly, 20 mL of blood placed in tubes containing ethylenediamine tetraacetic acid (EDTA) was diluted with an equal volume of PBS. Then, the PBMCs were isolated through density gradient centrifugation on Lymphoprep^TM^ (Alere Technologies AS, Oslo, Norway) at 400 × *g* for 30 minutes at 4°C. The interphase cells containing the PBMCs were collected and washed twice with PBS supplemented with 1% fetal bovine serum (FBS; Thermo Fisher Scientific), and then re-suspended in a complete Roswell Park Memorial Institute (RPMI)-1640 medium (Thermo Fisher Scientific) supplemented with 10% FBS, 100 U/mL penicillin G, 100 μg/mL streptomycin, and 0.25 μg/mL amphotericin B. Cells were seeded onto 96 wells-microtiter plates or 6 wells-microtiter plates (SPL Life Sciences, Gyeonggi-do, Korea) and cultivated at 37°C with 5% CO_2_, or centrifuged at 400 × *g* for 5 minutes to collect cell pellets. They were double immunofluorescent stained and either investigated under transmission electron microscopy or their cytokine mRNA expression was determined by quantitative reverse transcriptase-polymerase chain reaction (qRT-PCR), as described below. Cell pellets were fixed in 10% neutral buffered formalin and processed to obtain the FFPE samples. They were then investigated for apoptosis using the Terminal deoxynucleotidyl transferase-mediated dUTP nick end labeling (TUNEL) assay, as described below.

### Terminal deoxynucleotidyl transferase-mediated dUTP nick end labelling (TUNEL) assay

The TUNEL assay of the FFPE elephant tissues from the EEHV1A-HD, EEHV4-HD and EEHV-negative calves or PBMCs of the persistent EEHV4-infected and EEHV-negative calves was performed using an *In Situ* Cell Death Detection kit (Sigma Aldrich, St. Louis, MO, USA), according to the manufacturer’s instruction. Briefly, subsequent to being deparaffinized and rehydrated, slides were washed with PBS twice and microwaved for 2 minutes. Following a second round of washing with PBS, slides were incubated with fresh-prepared TUNEL reaction mixture for 1 hour at 37°C in a moist chamber. Subsequent to being washed twice with PBS, the nuclei were counter stained with bisbenzimide (Sigma Aldrich).Slides were then washed with PBS and observed under fluorescence microscopy. Percentage of apoptotic cells was determined by counting the fluorescent positive cells versus intact nuclei using the ImageJ (National Institutes of Health, Bethesda, MD), as described previously [6].

### Immunofluorescence

The elephant PBMCs were double immunofluorescent stained, as previously described [6]. Briefly, the cultures were fixed with 4% paraformaldehyde for 15 minutes at RT, and treated with 0.25% Triton X-100 in PBS (0.25% PBST) for 15 minutes. Then, the cells were incubated with 1% bovine serum albumin (BSA) in PBST for 30 minutes at RT, followed by incubation with specific primary antibodies diluted with 1% BSA in 0.25% PBST at 37°C for2 hours. The primary antibodies were rabbit polyclonal anti-EEHV gB (1:500), and mouse monoclonal anti-Iba-1 (1:400; Millipore Corporation). The secondary antibodies were incubated for 45 minutes at RT with a mixture of the Cy3–conjugated goat anti-mouse and FITC-conjugated goat anti-rabbit antibodies (all from Jackson ImmunoResearch, Suffolk, UK), at a dilution of 1:200. The nuclei were counterstained using bisbenzimide (Sigma Aldrich) for 10 minutes at RT. The cultures were analyzed and photos were taken under an inverted fluorescent microscope.

### Transmission electron microscope

To study the ultrastructure of the persistent EEHV4-infected and EEHV-negative elephant PBMCs, 20 mL of blood was acquired from the ear veins and the PBMCs were isolated, as described above. They were then prepared for electron microscopy, as previously described [21]. Briefly, cells were fixed in 2.5% glutaraldehyde for 48 hours, washed three times with PBS, and post fixed in 2% osmium tetroxide for 1 hour at RT. After being serially dehydrated in ethanol, the cells were incubated in propylene oxide and embedded in resin (EM-bed 812, Electron Microscope, WA, USA). Thereafter, they were cut, mounted on a copper grid, and electron contrasted with uranyl acetate and lead citrate. The grids were examined and photos were taken under a JEM-2200FS transmission electron microscope (JEOL Ltd., Tokyo, Japan).

### Quantitative RT-PCR and conventional PCR

The total RNA of PBMCs obtained from the persistent EEHV4-infected and EEHV-negative calves was extracted and determined using Nucleospin^®^ RNA II (Machery-Nagel GmbH, Dauren, Germany), as suggested by the manufacturer. The cDNA was synthesized from 50 ng total RNA with random primers and high capacity cDNA reverse transcriptase kits (Thermo Fisher Scientific). This study used the following oligonucleotide primer genes-TNF-α, IFN-γ, IL-1β, IL-2, IL-4, IL-8, IL-10, IL-12 and GAPDH, as described previously[22, 23].To quantify the expression of elephant cytokines, real time PCR (SensiFastSYBR^®^Hi-ROX Kit, Bioline, Luckenwalde, Germany) was performed on an ABI7300 thermocycler (Applied Biosystems, CA, USA) in a total reaction volume of 10 μL. The threshold cycles (Ct) of all genes were used to calculate gene expression by the 2^-ΔΔCT^ method [24], normalized to that of GAPDH genes and compared to the EEHV-negative control group. The data, obtained from triplicate wells from two independent experiments, were shown as mean fold changes ± standard errors. EEHV infection was determined using conventional PCR using the primers for polymerase and terminase genes, as previously described [25].

### Statistical analysis

A statistical analysis of the scores of immunolabeling positive cells, TUNEL-postive cells and qRT-PCR wasperformed using GraphPad Prism 5 (GraphPad Inc., La Jolla, CA, USA). Either Chi-square or t-test was performed, depending on the data type. In addition, the relationship between the scores of EEHV gB-positive cells and those of Iba-1-positive cells from the hearts, lungs, lymph nodes, intestines and salivary glands of calves from the EEHV1A-HD, EEHV4-HD, and co-infected EEHV1A and 4-HD groups, was analyzed using Spearman’s rank correlation test (R program version 3.5.1, R Foundation for Statistical Computing, Vienna, Austria). Statistical significance was designated as *p* ≤ 0.05.

## Results

### Comparison of blood leukogram in EEHV1A-HDand EEHV4-HD Asian elephant calves

Analysis of the blood obtained within the three days prior to an elephant’s death from either EEHV1A-HD or EEHV4-HD indicated marked neutrophilia in all EEHV-infected calves. Monocytopenia and thrombocytopenia were observed in some animals, especially those infected with EEHV1A (Table 2). In contrast, blood leukograms taken 2–3 weeks before death in the EEHV1A- and EEHV4-infected calves did not differ significantly from the reference values (data not shown).

**Table 2:** Blood clinical parameters obtained within three days prior to the animal’s death.

Parameter	EEHV1A-infected calves	EEHV4-infected calves	Reference range ^[^^26^^]^
Number of animals	3	2	-
Mean age (years old)	1.7	3.5	-
Mean pack cell volume (%)	35.3	41.5	30–40
Mean hemoglobin (g/dL)	12.7	14.85	11–15
Mean RBC count (x10^6^ cells/dL)	3.1	3.78	2.5–5
Mean MCV (fL)	114.6	109.2	80–160
Mean MCHC (g/dL)	36	36.15	25–40
Mean WBC count (cells/μL)	13,913.3	14,315	10,000–18,000
Mean neutrophil count (cells/μL)	7,110.3	7,595	2,000–4,000
Mean lymphocyte count (cells/μL)	5,626.7	4,482	5,000–8,000
Mean monocyte count (cells/μL)	1,353.7	2,132.5	2,000–4,000
Mean eosinophil count (cells/μL)	32.7	105.5	100–1,000
Mean basophil count (cells/μL)	-	-	rare
Mean platelet count (x10^3^ cells/μL)	48.7	228	200–600

### Infiltration of Iba-1-positive macrophages in inflamed tissues increased in EEHV-HD calves

**Fig 1:**
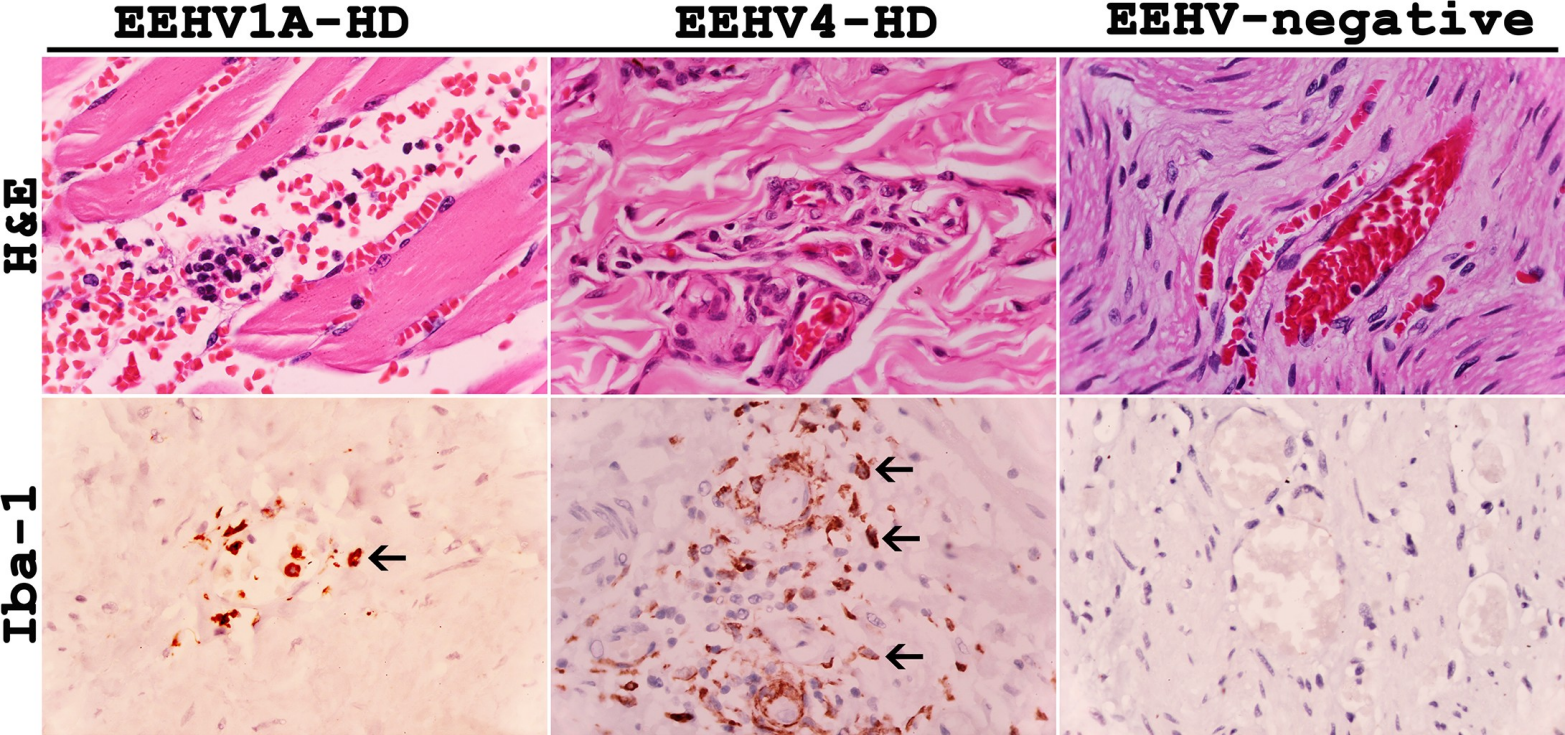
Histopathological and immunohistochemical labeling for Iba-1 of tissue samples from the EEHV1A-HD, EEHV4-HD and EEHV-negative calves. EEHV infections resulted in a non-suppurative and granulomatous vasculitis and perivasculitis, and increased extravasation of the Iba-1 positive cells (arrows) out of blood vessels of the EEHV-HD calves, when compared to the EEHV-negative control.

Histopathological analysis of the tissue samples from the EEHV1A-HD, EEHV4-HD, and co-infected EEHV1A and 4-HD calves revealed non-suppurative or granulomatous inflammations, especially in the perivascular areas of the heart, intestine, liver and lymph node, when compared to the EEHV-negative control animals (Fig 1). Despite observing severe edema and hemorrhaging in most of the internal organs of those calves that died from EEHV-HD, they exhibited only mild to moderate degrees of vascular and perivascular inflammations (Fig 1). These findings suggested that the pathogenesis of EEHV-HD involves not only the inflammation and rupture of vascular endothelia that cause leakage of proteins, fluid or cells from blood circulation, but also cellular mediators that may enhance vascular permeability.

**Fig 2:**
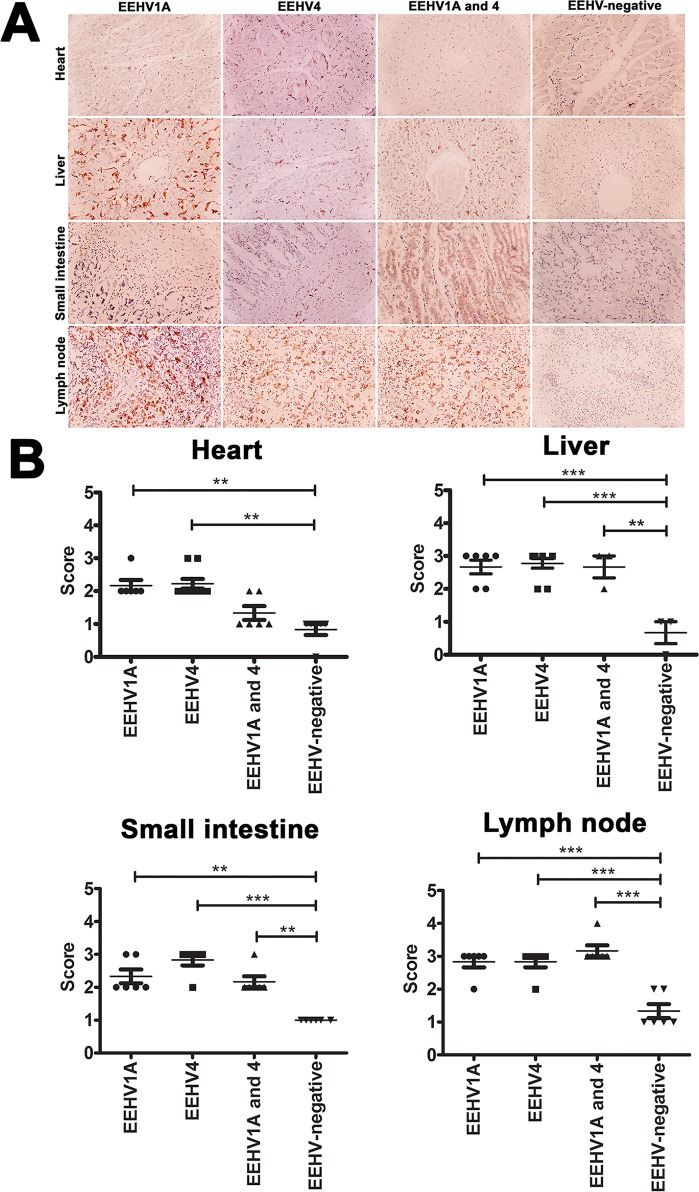
Immunohistochemical labeling and scoring of Iba-1 positive cells in tissues of the EEHV1A-HD, EEHV4-HD, co-infected EEHV1A and 4-HD, and EEHV-negative calves. Significant immunolabeling of Iba-1 antibodies was observed within the blood vessels and parenchymal tissues of various internal organs of EEHV-infected calves, including the hearts, livers, small intestines and lymph nodes, compared to the EEHV-negative control group (**A, B**). Scoring was obtained from three independent observers and data presented as a mean ± standard error. Asterisks indicate statistical significance (*p<0.05, **p<0.01, ***p<0.001), compared to the EEHV-negative control group.

Immunohistochemical labeling for Iba-1 of elephant tissues that died in association with EEHV1A-HD, EEHV4-HD, or co-infections of EEHV1A and 4-HD indicated a significant increase in Iba-1 positive cells in the hearts, livers, small intestines and lymph nodes, when compared to those in the EEHV-negative control (Fig 2A and 2B). In addition, Iba-1 immunolabeling positive cells increased significantly in the salivary glands, lungs, kidneys and large intestines of the EEHV4-HD calves compared to the samples of EEHV-negative calves (S1 Fig). No significant amount of Iba-1 positive cells was observed in the spleens or stomachs of the EEHV1A-HD, EEHV4-HD, or EEHV1A and 4-HD infected calves, when compared to the EEHV-negative calves (S2 Fig).

### Correlation between EEHV gB-positive cells and Iba-1-positive cells in the elephant tissues of EEHV1A-HD, EEHV4-HD, and co-infected EEHV1A and 4-HD calves

**Fig 3:**
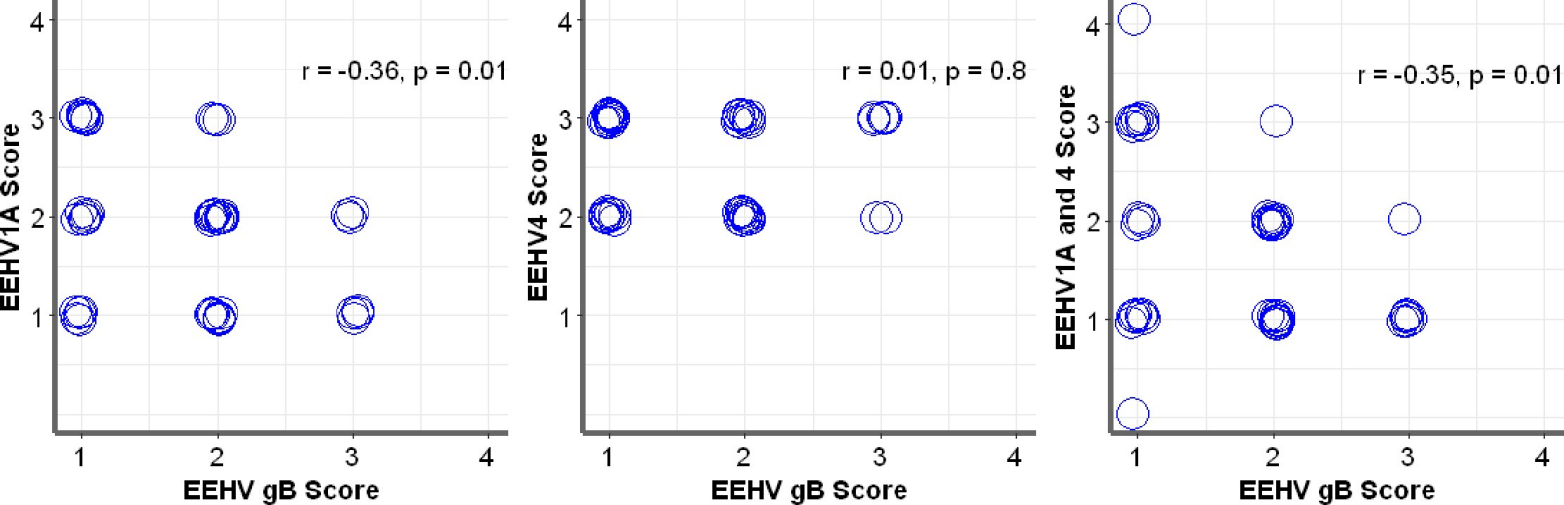
Correlative scoring of EEHV gB-positive and Iba-1-positive cells using Spearman’s rank correlation test. Immunolabeling positive cells of the EEHV gB and Iba-1 antibodies from the hearts, lungs, livers, lymph nodes, kidneys, salivary glands, and intestines were scored by three independent observers. Scoring of EEHV gB positive and Iba-1positive cells in the EEHV1A-HD and co-infected EEHV1A and 4-HD calves revealed a significant negative correlation; the Iba-1 positive cells in the tissues increased while only a low level of EEHV gB positive cells were detected. No significant correlation was observed in the EEHV4-HD calves.

Analysis of the scores of EEHV gB and Iba-1-positive cells from the hearts, lungs, livers, kidneys, salivary glands, lymph nodes, and intestines demonstrated a significant negative correlation between these two antigens in EEHV1A-HD (r = -0.36) and co-infected EEHV1A and 4-HD (r = -0.35) calves, while no significant correlation was identified in the EEHV4-HD calves (Fig 3). These results suggested that Iba-1 positive monocytes from blood vessels infiltrated the inflamed tissues of the internal organs, despite the fact that fewer EEHV gB antigens were detected. Therefore, it is possible that the EEHV1A infection and co-infections of EEHV1A and 4 in elephant blood leukocytes or tissues induce expression of chemoattractive mediators that might play a role in the movement of monocytes out of the blood circulation. Hence, immunolabeling of EEHV gB was low, while immunolabeling for Iba-1 was high. On the other hand, despite a clear increase in Iba-1 positive cells in the internal organs of EEHV4-HD calves, the EEHV4 gB immunolabeling positive cells observed in the tissues of EEHV4-HD calves also increased (Fig 3). These results suggest differences in the pathological mechanisms of the different EEHV subtypes in Asian elephants.

### Apoptosis of the peripheral blood monocytes and inflamed tissues was high in the EEHV1A-HD calves

**Fig 4:**
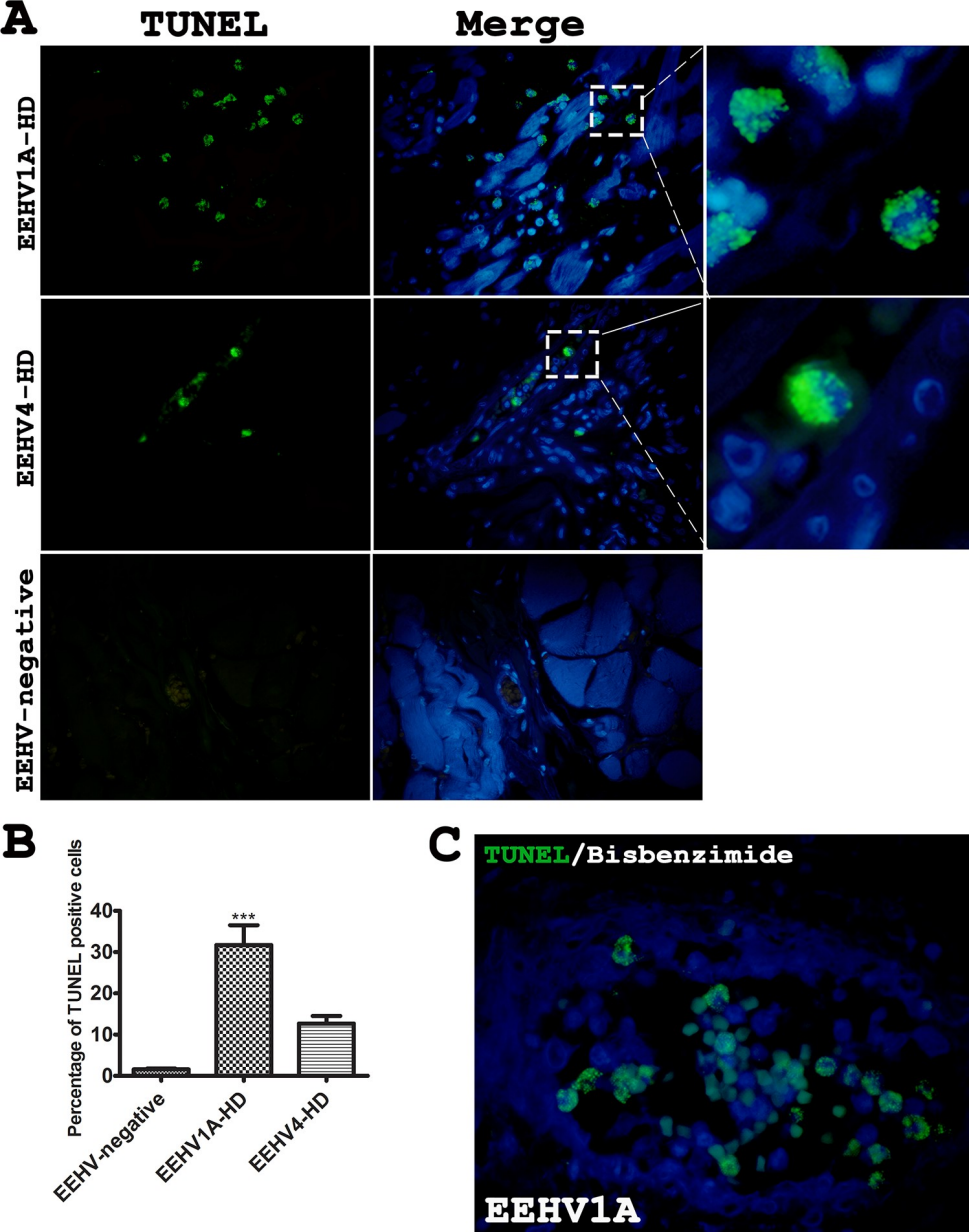
Terminal deoxynucleotidyl transferase-mediated dUTP nick end labelling (TUNEL) assay of the tissues from the EEHV1A-HD, EEHV4-HD and EEHV-negative calves. TUNEL assay revealed remarkable and significant apoptosis in the EEHV-HD calves (**A, B**). Apoptosis in the EEHV1A-HD calves was predominantly seen in the mononuclear cells within the blood vessels, compared to the EEHV-negative control (**C**). Data presented as mean ± standard error. Asterisks indicate statistical significance (***p<0.001) compared to the EEHV-negative control group.

Terminal deoxynucleotidyl transferase-mediated dUTP nick end labeling (TUNEL) assay of FFPE tissues from the heart, spleen and intestine of the EEHV1A-HD, EEHV4-HD and EEHV-negative calves revealed significant apoptosis in the EEHV1A-HD calves, but not the EEHV4-HD calves, compared to the EEHV-negative control (Fig 4). TUNEL positive cells were high, up to 30% of the nucleated cells, in the EEHV1A-HD cases compared to the EEHV-negative control (Fig 4C). The increase in apoptotic cells in the EEHV1A-HD cases was mainly observed in the mononuclear cells in the blood vessels. Blood analysis of the EEHV1A-infected calves three days prior to death indicated monocytopenia compared to the reference value (Table 2). These findings suggested that apoptosis of the monocytic cells may be associated with the decreased monocytes observed in the blood leukograms of the EEHV1A-HD calves. TUNEL assay of the persistent EEHV4-infected elephant PBMCs showed no significantly difference of the positive cells when compared to the EEHV-negative calves (S3 Fig).

### Localization of EEHV gB antigen in the PBMCs of persistent EEHV4-infected calves

**Fig 5:**
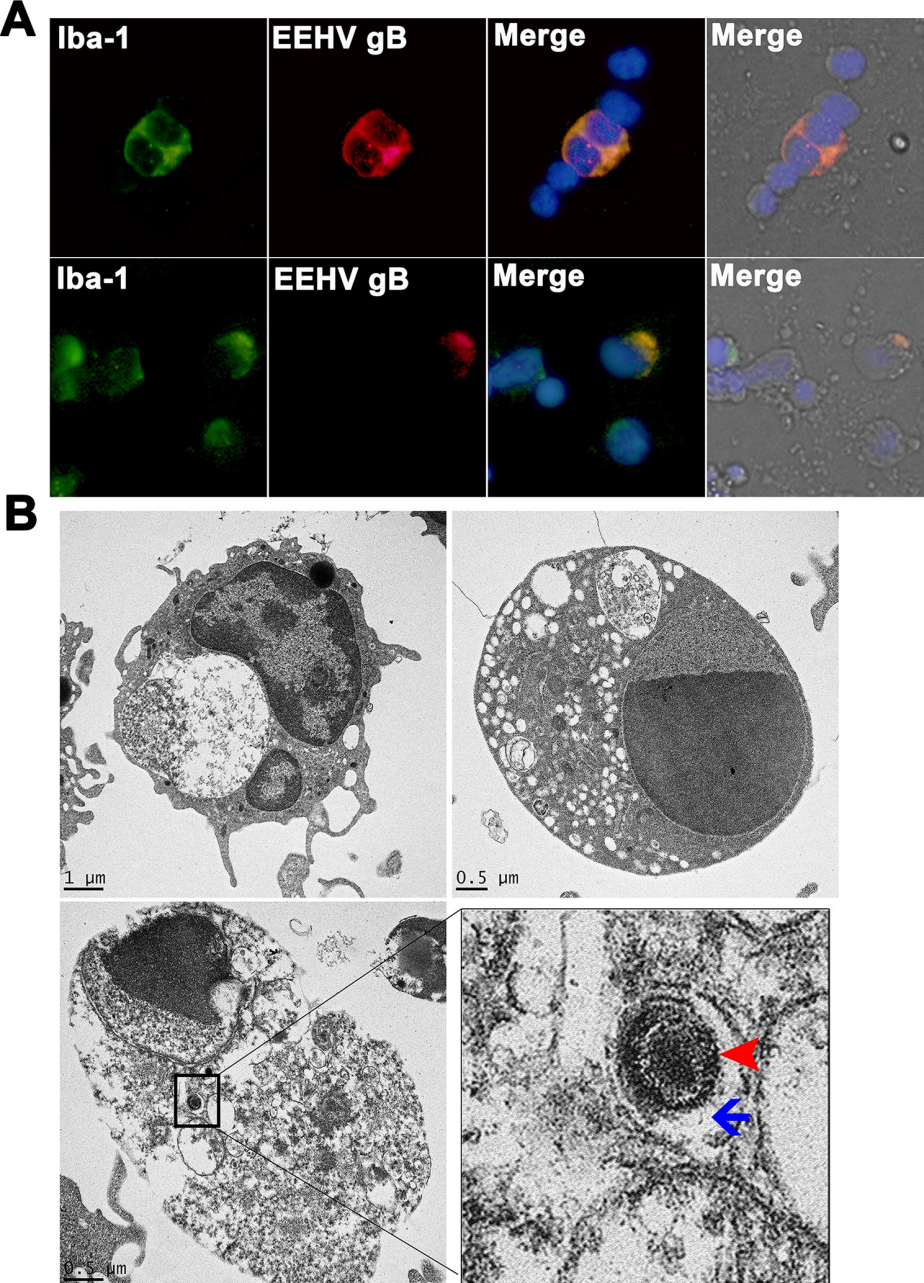
Immunofluorescence and electron micrograph of the persistent EEHV4-infected elephant PBMCs. PBMCs were obtained from healthy elephants known to be infected with EEHV4 for at least six months. PBMCs were then either double immunofluorescent stained with anti-EEHV gB and anti-Iba-1 antibodies (**A**) or processed for transmission electron microscopy (**B**). Staining of elephant PBMCs with anti-EEHV gB (red) revealed antigen distribution in the cytoplasm of the Iba-1 positive cells (green), suggesting their monocytic phenotype (**A**). The EEHV infection was observed in both non-degenerated and degenerated Iba-1 positive cells. Transmission electron micrographs of the EEHV4-positive elephant monocytes revealed segmented nuclei and variable degrees of vacuolation in the cytoplasm, with some endosomes containing virus particles (inset, **B**). Degenerated monocytes were observed through chromatin and cytoplasmic lysis. Enveloped viruses (arrowheads) with a diameter of ~150 nm were observed in the cytoplasmic endosomes (arrows) of degenerated cells (**B**).

To investigate the impact of persistent EEHV infection in elephant PBMCs, blood obtained from elephants with a previous history of EEHV4 DNAemia was analyzed by double immunofluorescence and electron microscopy. The results demonstrated that the EEHV gB antigens were mainly located in the cytoplasm of the Iba-1 positive monocytic cells (Fig 5A). Most EEHV gB positive cells were also positive for Iba-1 immunolabeling. Ultrastructural analysis of persistent EEHV4-infected elephant PBMCs revealed that EEHV4 infection in elephant monocytes was non-productive and caused moderate degeneration of the infected cells (Fig 5B). Virus-infected monocytes displayed central chromatin lysis, expansion and aggregation of nucleoli. Mitochondria and ribosomes were degenerated and large phagocytic vacuoles were found (Fig 5B). Viral particles (arrowhead) were seen within the endosomes (arrow) of the EEHV4-infected PBMCs (Fig 5B).

### Cytokine mRNA expression in the persistent EEHV4-infected elephant PBMCs

**Fig 6:**
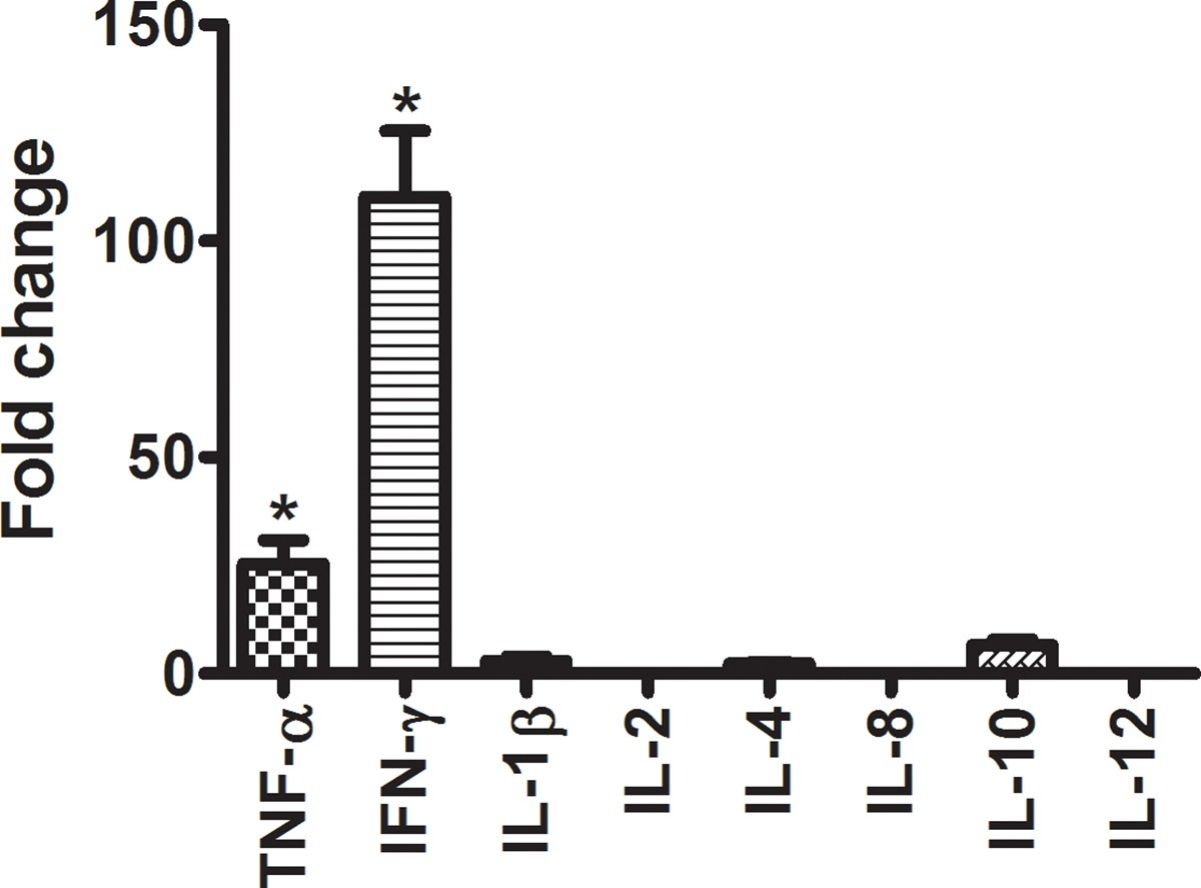
Upregulation of cytokine mRNA expression in the persistent EEHV4-infected calves. Quantification of cytokine mRNA expressions of elephant PBMCs revealed significant up-regulations of TNF-α and IFN-γ in the persistent EEHV4-infected calves compared to the EEHV-negative controls. Data represent the mean ± standard error from two independent experiments. Asterisks indicate statistically significant differences (*p<0.05) compared to the EEHV-negative controls.

Quantification of cytokine mRNA expressions of the persistent EEHV4-infected and EEHV-negative elephant PBMCs revealed significant up-regulations of TNF-α and IFN-γ mRNA expressions in the persistent EEHV4-infected calves compared to the EEHV-negative control group (Fig 6). Expressions of mRNA of IL-1β, IL-2, IL-4, IL-8, IL-10 and IL-12 genes in the persistent EEHV4-infected calves were non-significant, compared to the EEHV-negative control group (Fig 6).

## Discussion

In cases of EEHV infections in Asian elephants, a sudden reduction of monocytes in blood circulation, known as monocytopenia, is one of the prognostic hematological parameters of EEHV-HD [5–9]. Despite some studies observing EEHV genomes and antigens in the monocytes/macrophages of EEHV-HD calves, and postulating that these cells are carriers for virus dissemination [6, 19], the exact roles the monocytes/macrophages play in cases of either acute infection and subsequent fatality or persistent EEHV-infection are unclear. Moreover, it is not known why monocytopenia has been observed in some cases of fatal EEHV-HD infection [5, 6, 9], or in some episodes during persistent EEHV infection [7, 8]. The present study observed for the first time apoptosis of the peripheral blood mononuclear cells in acute cases of fatal EEHV-HD infection and in persistent EEHV4 infections. Moreover, phagocyted apoptotic cells, together with increased trafficking of blood monocytes to the inflamed tissues, may cause monocytopenia in the cases of acute or persistent EEHV infection in Asian elephants. Furthermore, this study has shown that the virus-laden blood mononuclear cells likely serve as vehicles for disseminating EEHV, rather than as a viral reservoir, during both acute and persistent EEHV infections in Asian elephants.

Originating from the bone marrow progenitor cells, monocytes are trafficked via the blood stream to the peripheral tissues where they differentiate into macrophages or dendritic cells, and then play an essential role in immune defense mechanisms [15, 17, 18, 27–29]. However, monocytes/macrophages also contribute to bystander tissue destruction during some infections and inflammatory diseases [15, 29]. The magnitude of monocyte/macrophage infiltration in inflamed tissues can, therefore, be used as a marker for determining severity of the lesions [15, 17, 30]. Previous studies have shown that viruses, such as porcine reproductive and respiratory syndrome virus (PRRSV) and feline infectious peritonitis virus (FIPV), infect monocytes/macrophages and cause their necrosis and apoptosis, both *in vivo* and *in vitro* [31–34]. Apoptosis is a form of programmed cell death characterized by decreased nuclear and cellular volume, nuclear fragmentation, and plasma membrane blebbing, leaded to cell fragments called apoptotic bodies; it can be classified into extrinsic and intrinsic pathways [35, 36]. There can be substantial crosstalk between the intrinsic and extrinsic pathways, triggered in response to activation of Toll-like receptors (TLRs), which usually result from viral infections [35, 37–39]. Monocytes are non-dividing cells with a short half-life that circulate in the blood and make viral replication difficult or almost impossible [40]; as such, apoptosis of peripheral blood monocytes has been suggested to be one of the defense mechanisms against viral infection [41, 42]. In the present study, ultrastructural photomicrographs of EEHV viral particles in peripheral blood monocytes of persistent EEHV4-infected calves and positive signals of TUNEL assay in monocytes of the EEHV-HD calves suggest that EEHV infection of monocytes seems to be non productive and result in apoptosis of the EEHV-infected cells. Non productive infection and apoptosis of the peripheral blood monocytes has been observed in infection with other betaherpesviruses, such as human cytomegalovirus (HCMV) [43, 44]. Infection of peripheral blood monocytes with HCMV leads to induction of anti-apoptotic proteins, such as myeloid cell leukemia-1 (Mcl-1) and B cell lymphoma-1 (Bcl-1), as causes delay of apoptosis in the infected cells [12, 45, 46]. The delay apoptosis of HCMV-infected monocytes allow these cells to bridge the 48–72 hours viability gate, so then they can differentiate into mature phenotypes required for HCMV persistence, such as macrophages or dendritic cells [12, 47]. In contrast to the present study, apoptosis of the peripheral blood mononuclear cells was predominantly observed in the fatal cases of EEHV1A-HD and EEHV4-HD infections and in a lower extent of persistent EEHV4-infection. Despite these findings may support the hypothesis indicating the functional role of monocytes in inhibiting the viral replication, as seen in the infection of dengue virus, PRRSV and FIPV [10, 11, 33, 34], it remains unknown whether infection of EEHV in monocytes causes delay apoptosis. Moreover, since positive signals of TUNEL assay were also observed in the cytoplasm of peripheral blood monocytes, it is possible that in addition to the apoptotic bodies that seen in EEHV-infected monocytes, apoptotic bodies derived from the EEHV-infected cells may also had phagocytosed by the phagocytic cells, such as monocytes. Then, these cells may serve as carrier for virus dissemination to other organs. However, to define precise pathomechanisms of apoptosis in the EEHV-infected elephant cells, further studies are required.

The present study showed that up-regulation of cytokines, including TNF-α and IFN-γ, by peripheral blood mononuclear cells during persistent EEHV infections may play a role in the pathomechanism of diseases caused by EEHV. TNF-α related apoptosis inducing ligand (TRAIL), a type II transmembrane protein belonging to the TNF superfamily, has been shown to contribute to the induction of apoptosis in several types of tumor or virus-infected cells [35, 48, 49]. Moreover, TNF-α has been shown to enhance vascular hyperpermeability in dengue hemorrhagic fever [27, 50]. This study showed that TNF-α mRNA up-regulated in the persistent EEHV4-infected calves suggesting that during EEHV infection, TNF-α not only contributed to apoptosis of the EEHV-infected cells, but also enhanced transmigration of leukocytes out of the blood vessels. Thus, it is reasonable to postulate that migration of peripheral blood monocytes out of the blood vessels is also associated with the monocytopenia observed in the EEHV-infected elephants. We would like to stress the fact that monocytopenia observed in the EEHV-HD cases was not due to the homing of monocytes/macrophages in the spleen since the Iba-1 immunolabeling positive cells in the spleen was not differed between the EEHV-HD cases and the EEHV-negative controls. Meanwhile, we did not explore the decrease of monocytic production in bone marrow in the present study. Thus, it remains unclear if EEHV infections possess an impact on monocytic production in bone marrow. Though, since studies have suggested that recruitment of monocytes from peripheral blood vessels into tissue parenchyma might provide a pathway for pathogen dissemination, such as observed in the HCMV infection [28, 51], it is likely that while EEHV circulates in the blood stream by infecting monocytes, it gets into the target organs by infecting or transmigrating through vascular endothelia, as shown by viral inclusions in the endothelial cells of some infected tissues.

The increased up-regulation of IFN-γ mRNA expressions in persistent EEHV4-infected elephant PBMCs observed in the present study indicated that type 1 interferon may play a role in inhibiting reactivation of EEHV during persistent EEHV infections. Studies have shown that IFN-γ attenuates viral infections in humans and animal models [52–54]. However, despite a previous report that showed that IFN-γ response in the latent EEHV-infected elephants were largely from the CD4^+^ T lymphocytes, and were prone to respond to EEHV antigens [55], it remains unclear whether CD4^+^ T lymphocytes are also targeted by EEHV. The up-regulation of IFN-γ mRNA expression in the persistent EEHV4-infected elephant PMBCs that this study observed further demonstrated the important role of type 1 interferon during persistent EEHV infections in Asian elephants.

This study had some limitations. First, we have only small sample size of the EEHV-HD cases. Hence, further study may be required to support the hypotheses proposed in the present study. Second, infection of elephant blood monocytes with EEHV could not be investigated using cell cultures, as the virus has yet to be successfully cultivated. Third, using both immunofluorescene and immunohistochemistry, we failed to demonstrate whether T and B lymphocytes were targeted by the EEHV. In summary, the present study hypothesized that dissemination of EEHV through the target organs occurs via virus-laden blood monocytes, and that the dissemination of the virus is inhibited by host defense mechanisms, including apoptosis in the EEHV-infected cells and induction of type 1 interferon. Moreover, apoptosis, together with increased trafficking of blood monocytes to the inflamed tissues, may cause monocytopenia in the cases of acute or persistent EEHV infection in Asian elephants.

## Supporting information

S1 Fig Immunohistochemical and scoring of Iba-1 positive cells in tissues from the EEHV1A-HD, EEHV4-HD, co-infected EEHV1A and 4-HD, and EEHV-negative calves. Significant immunolabeling of Iba-1 antibodies were observed in various internal organs of EEHV4-HD calves, including the salivary glands, lungs, kidneys and large intestines, compared to the EEHV-negative control group (**A, B**). Scoring was obtained from three independent observers and data presented as a mean ± standard error. Asterisks indicate statistical significance (**p<0.01, ***p<0.001), compared to the EEHV-negative control group.
(TIF)

S2 Fig Immunohistochemical and scoring of Iba-1 positive cells in tissues from the EEHV1A-HD, EEHV4-HD, co-infected EEHV1A and 4-HD, and EEHV-negative calves. No significance of Iba-1 immunolabeling was observed in the stomachs and spleens of the EEHV1A-HD, EEHV4-HD, or co-infected EEHV1A and 4-HD calves, when compared to the EEHV-negative control group.
(TIF)

S3 Fig TUNEL assay of the persistent EEHV4-infected elephant PBMCs. No significance of the TUNEL positive cells was observed in the EEHV4-infected PBMCs, when compared to the EEHV-negative control animals.
(TIF)
