# Supplementary figures and images for: Possible roles of monocytes/macrophages in response to elephant endotheliotropic herpesvirus (EEHV) infections in Asian elephants (Elephas maximus)
Source: PLoS One. 2019 Sep 6;14(9):e0222158. doi: 10.1371/journal.pone.0222158 (PMC6730851; doi:10.1371/journal.pone.0222158)

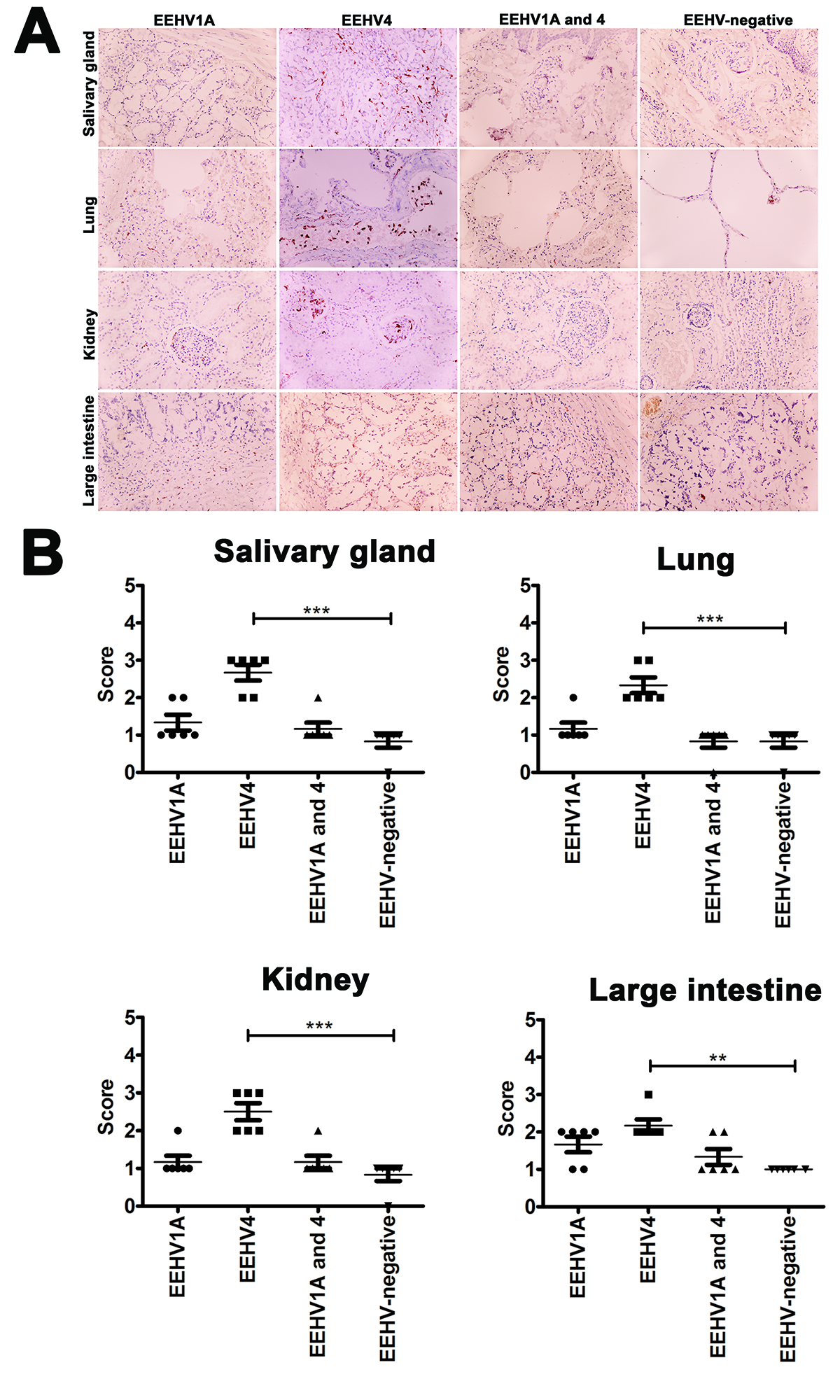

Supplement: S1 Fig — Significant immunolabeling of Iba-1 antibodies were observed in various internal organs of EEHV4-HD calves, including the salivary glands, lungs, kidneys and large intestines, compared to the EEHV-negative control group (A, B). Scoring was obtained from three independent observers and data presented as a mean ± standard error. Asterisks indicate statistical significance (**p<0.01, ***p<0.001), compared to the EEHV-negative control group. (TIF) [file pone.0222158.s001.tif]

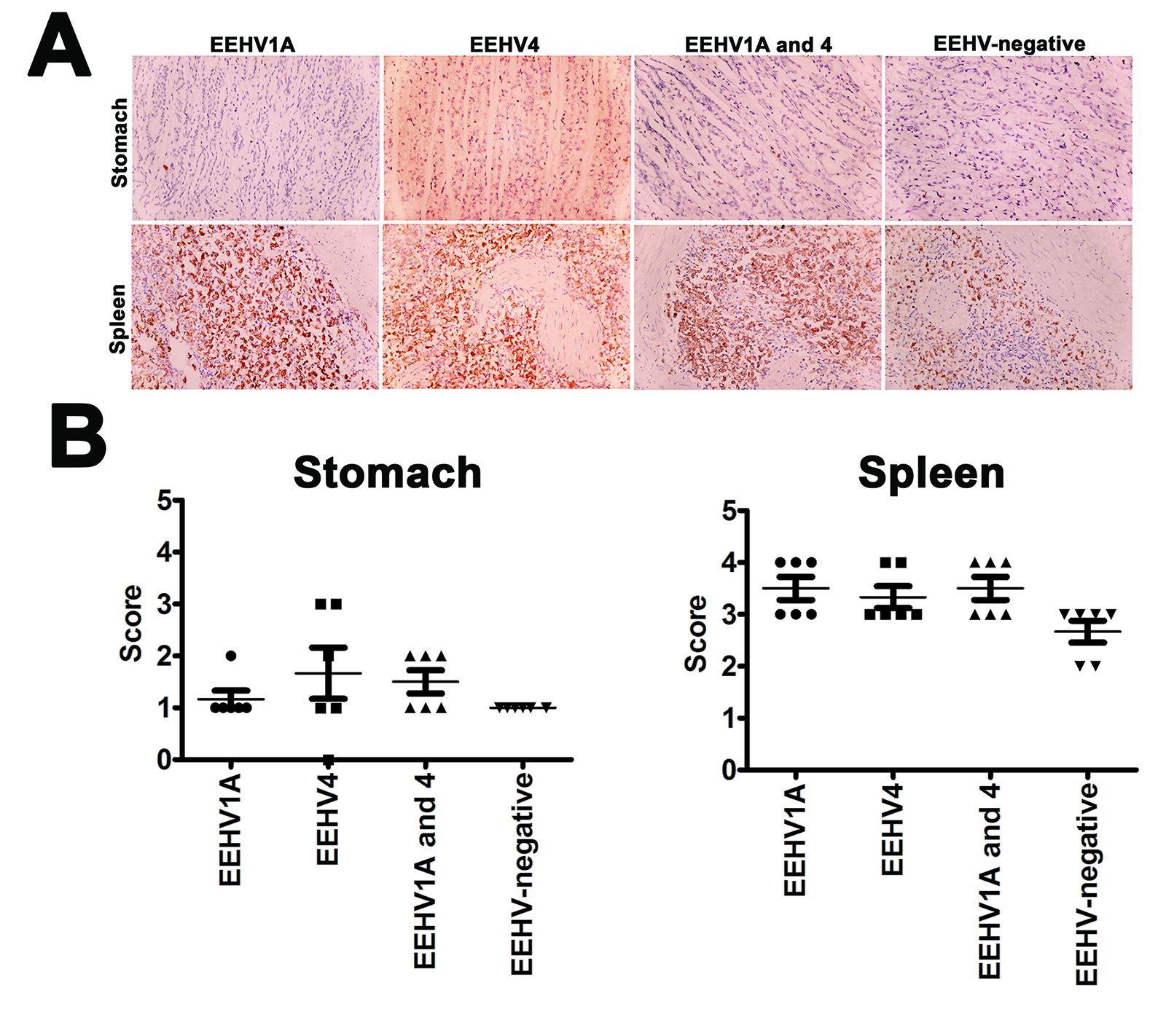

Supplement: S2 Fig — No significance of Iba-1 immunolabeling was observed in the stomachs and spleens of the EEHV1A-HD, EEHV4-HD, or co-infected EEHV1A and 4-HD calves, when compared to the EEHV-negative control group. (TIF) [file pone.0222158.s002.tif]

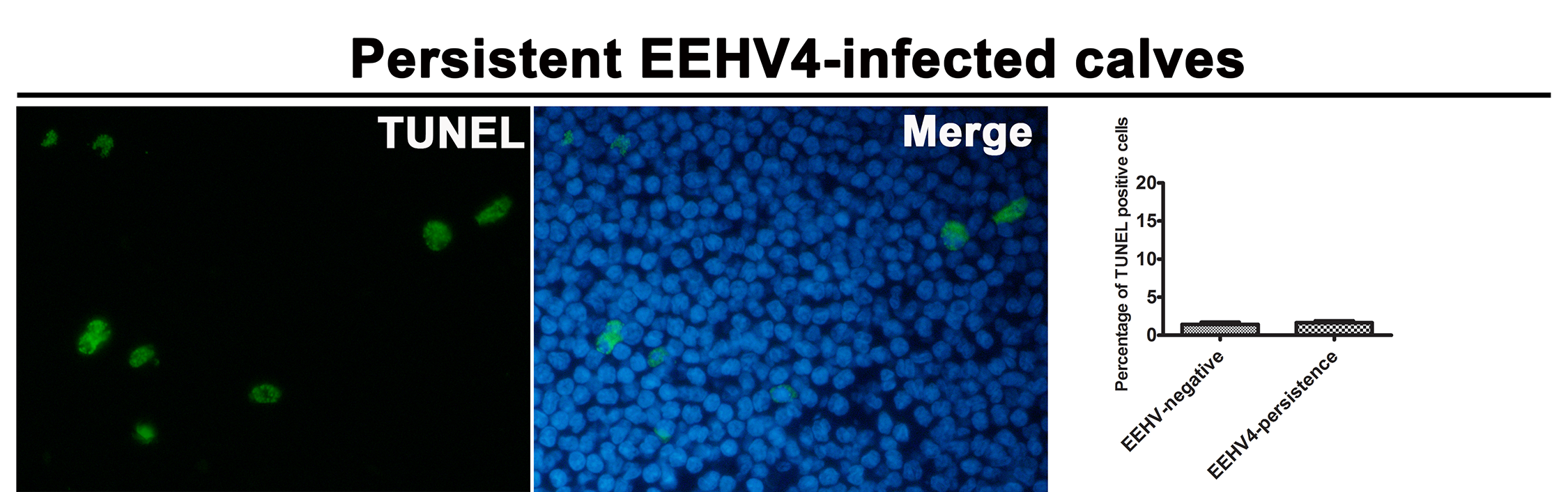

Supplement: S3 Fig — No significance of the TUNEL positive cells was observed in the EEHV4-infected PBMCs, when compared to the EEHV-negative control animals. (TIF) [file pone.0222158.s003.tif]
